# Lethal effect of blue light on Asian tiger mosquito, *Aedes albopictus* (Diptera: Culicidae)

**DOI:** 10.1038/s41598-022-14096-y

**Published:** 2022-06-16

**Authors:** Katsuya Taniyama, Masatoshi Hori

**Affiliations:** Graduate School of Agricultural Science, Tohoku University, Sendai, Miyagi 980-8572 Japan

**Keywords:** Zoology, Diseases, Optics and photonics

## Abstract

In our previous studies, we found that blue light has a lethal effect on various insect species and demonstrated that the most effective wavelength to control the hygiene pest, the mosquito, *Culex pipiens* form *molestus* (Diptera: Culicidae), is ~ 420 nm through all developmental stages. The genera *Aedes* and *Culex* include many globally crucial hygiene pest species that transmit serious diseases to humans and animals. However, effective lethal wavelengths have been shown to differ among insect species. In this study, we investigated the lethal effects of blue light on the Asian tiger mosquito, *Aedes albopictus*, using light-emitting diodes. Blue-light irradiation had a lethal effect on the larvae, pupae, and adults of *Ae. albopictus*. In particular, the 417-nm blue-light wavelength had a strong lethal effect on the larvae, showing 100% mortality before pupation at the photon flux density of 10 × 10^18^ photons·m^−2^·s^−1^. In contrast, no blue-light wavelength had a lethal effect on the eggs. Moreover, the 417-nm wavelength had the strongest effect on the pupae among the tested blue-light wavelengths. Our findings indicate that ~ 420 nm is the most promising blue-light wavelength to control populations of *Ae. albopictus* and *C. pipiens* f. *molestus*.

## Introduction

In our previous studies, we have found that irradiation with blue light (400–500 nm) has a lethal effect on various insect species^1,2^. This effect is considered to be useful for the development of clean and safe pest control techniques. In fact, light-emitting diode (LED) devices that emit blue light have recently begun to be applied to control insect pests in some food-processing facilities^3^. Therefore, we investigated the applicability of blue light toxicity to insects for reducing mosquito populations.

Mosquitoes are one of the most dangerous animals because their bites not only cause skin irritation but also may spread a variety of deadly diseases. In addition, for most of the arboviral diseases transmitted by mosquitoes, there is no preventive vaccine or medicinal treatment^4^. Therefore, mosquito-vector controls play a key role in reducing disease transmission^5^. Larvicides that can kill immature stages of mosquitoes are used to reduce adult mosquito populations. However, the use of larvicides has certain disadvantages, such as the development of insecticide resistance and disruption of the aquatic ecosystem. Therefore, eco-friendly control techniques that can kill immature stages of mosquitoes need to be developed.

In mosquitoes, we have previously demonstrated the lethal effect of blue light on *Culex pipiens* form *molestus*^1,6^*.* The mosquito vectors mainly belong to three genera, *Aedes*, *Anopheles*, and *Culex*^7^. In *C. pipiens* f. *molestus*, the most toxic blue-light wavelength is ~ 420 nm through all developmental stages^6^. However, in insects, effective blue-light wavelengths are species- and growth stage-specific^1,2,8^. Therefore, in this study, we investigated blue-light wavelengths highly toxic to each developmental stage of the Asian tiger mosquito, *Aedes albopictus*.

*Aedes* mosquitoes, especially those belonging to two species, i.e. *Ae. aegypti* and *Ae. albopictus*, bear relevance to human public health as vectors of many viral diseases including dengue, Zika, chikungunya, yellow fever, and Rift Valley fever^9^. The global distribution of dengue fever, particularly, has rapidly grown in recent decades, with approximately 390 million people estimated to be infected each year^10,11^. In addition, climate change is expected to exacerbate the risk of the global expansion of *Aedes*-borne virus transmission^12–18^.

*Aedes albopictus* originally originated from Southeast Asia, China, and Japan but has expanded its habitat to Europe, Western Africa, Southern Africa, and North and South America in the last 3–4 decades^19–22^. It is a vector of at least 26 diseases, including the above-mentioned diseases^23–27^. In Japan, the annual number of imported dengue cases has increased over the years, reaching 200 in recent years^28^. Tokyo experienced an outbreak of autochthonous dengue fever transmitted by *Ae. albopictus* in 2014. Therefore, a pyrethroid insecticide against adult mosquitoes and an insect growth regulator against larval mosquitoes were applied in the parks where mosquitoes were inspected for dengue virus presence^29^. In addition, water drainage from ponds and rainwater inlets and fountain cleaning in parks have been carried out every year since then^29^. However, insecticide resistance has developed in some mosquito species^30–32^, partly because of the expression of the knockdown resistance (*kdr*) gene that prevents the binding of pyrethroids to the sodium channel^33^. *Aedes albopictus* populations expressing the *kdr* gene have been found in Singapore, China, and the United States^33,34^. The widespread use of insecticides has led to the spread of *kdr* mutations in mosquito populations^35^.

In this study, we demonstrated the application of a blue-light wavelength for killing *Ae. albopictus* as a promising alternative to insecticide application, which can reduce the populations of *Aedes* mosquitoes, major arbovirus vectors, using light. It has been revealed that ~ 440- and ~ 470-nm blue lights show higher toxicity to *D. melanogaster* than that at ~ 380-nm UVA^1^. Moreover in *C. pipiens* f. *molestus*, the toxicity of ~ 420 nm blue light is shown to be higher than that of ~ 380 nm UVA^6^. Therefore, in this study, we also investigated the toxicity of ~ 380-nm UVA to *Ae. albopictus* for comparison.

## Result

### Lethal effect of blue-light irradiation on Ae. albopictus eggs

**Figure 1:**
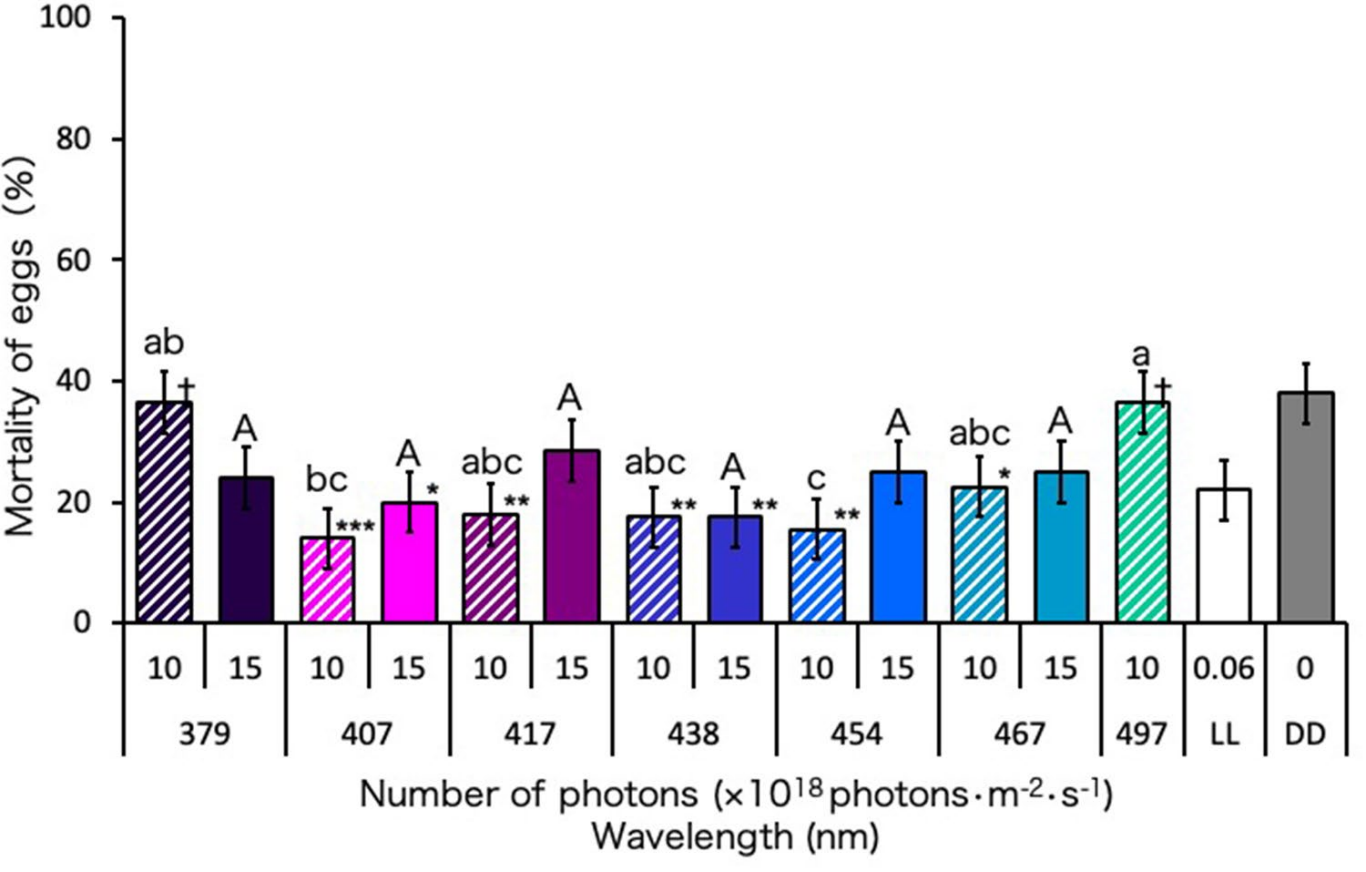
Mortality of *Aedes albopictus* irradiated with blue light during the egg stage. Data represent the means ± standard errors. Asterisks above the bars indicate significant differences between the treatments (UVA and blue light irradiation) and control [dark condition (DD)] (Steel test: *p < 0.05, **p < 0.01, ***p < 0.001). Daggers above the bars indicate significant differences between the treatments (UVA and blue light irradiation) and the control [continuous white light condition (LL)] (Steel test: ^†^*p* < 0.05). Bars with the same letters are not significantly different (Steel–Dwass test, *p* > 0.05). Ten replications (20 eggs per replicate) were conducted.

The mortalities of *Ae. albopictus* eggs continuously irradiated with blue-light wavelengths of 407, 417, 438, 454, and 467 nm at 10 × 10^18^ photons·m^−2^·s^−1^ were significantly lower than the mortalities of those kept under dark conditions (DD) (Fig. 1). However, the mortalities of the eggs irradiated with 417, 454, and 467 nm at 15 × 10^18^ photons·m^−2^·s^−1^ did not significantly differ from the mortalities of those kept under DD. Continuous irradiation with the wavelengths of 379 nm (UVA) and 497 nm at 10 × 10^18^ photons·m^−2^·s^−1^ resulted in egg mortalities similar to those obtained under DD. The egg mortalities under continuous white light condition (LL) did not significantly differ from those irradiated with all wavelengths of light tested, except for 379 and 497 nm at 10 × 10^18^ photons·m^−2^·s^−1^ (Fig. 1).

### Lethal effect of blue-light irradiation on the larval to pupal stages of Ae. Albopictus

**Figure 2:**
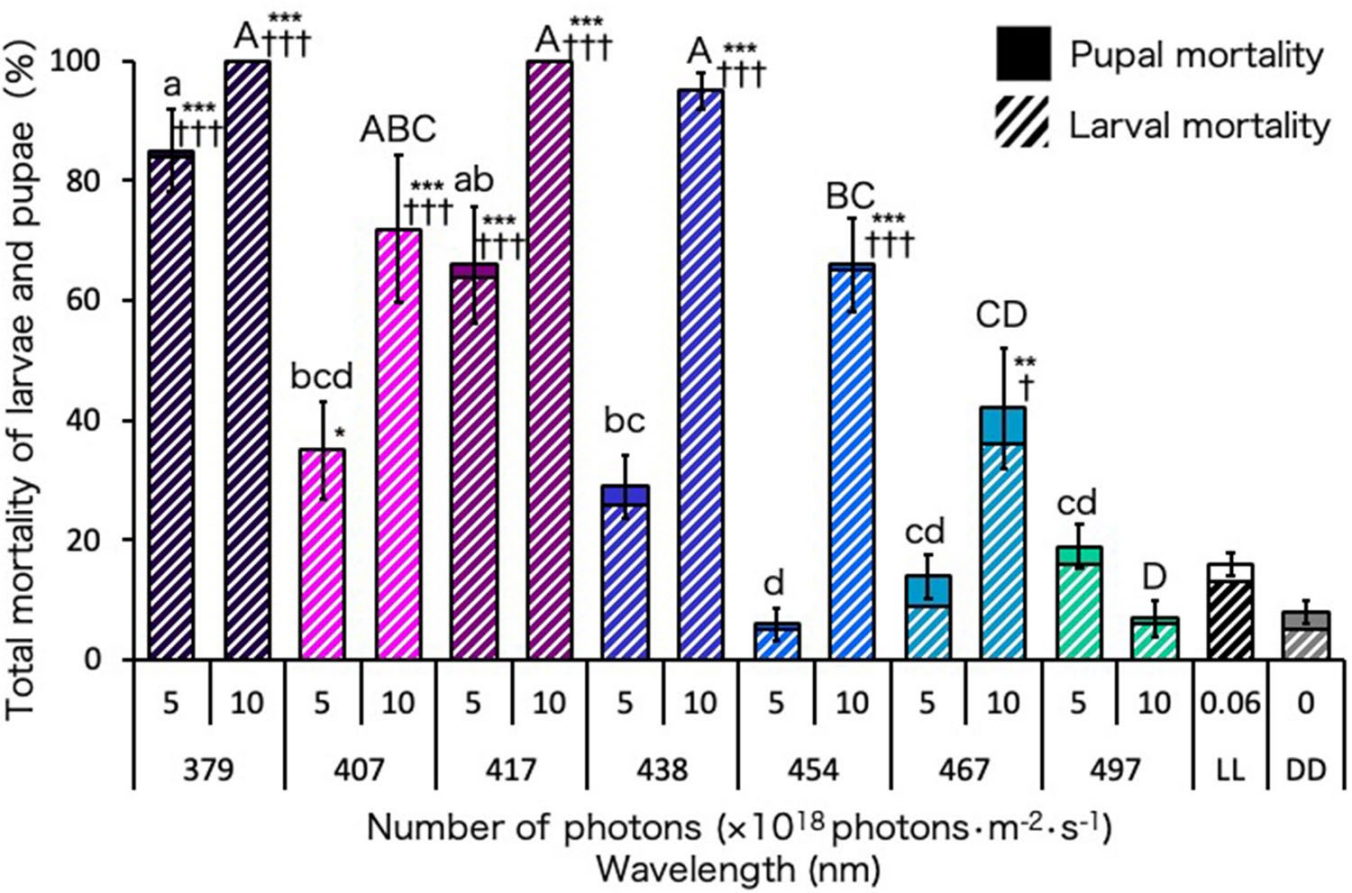
Mortality of *Aedes albopictus* irradiated with blue light during the larval to pupal stages. Data represent the means ± standard errors. Asterisks above the bars indicate significant differences between the treatments (UVA and blue light irradiation) and control [dark condition (DD)] (Steel test: *p < 0.05, **p < 0.01, ***p < 0.001). Daggers above the bars indicate significant differences between the treatments (UVA and blue light irradiation) and the control [continuous white light condition (LL)] (Steel test: ^†^*p* < 0.05, ^†††^*p* < 0.001). Bars with the same letters are not significantly different (Steel–Dwass test, *p* > 0.05). Ten replications (10 larvae per replicate) were conducted.

Continuous irradiation with 379-nm UVA light and 407- and 417-nm blue light at a photon density of 5 × 10^18^ photons·m^−2^·s^−1^ during the larval to pupal stages significantly increased the total mortalities of larvae and pupae compared with those under DD (Fig. 2). In particular, at 5 × 10^18^ photons·m^−2^·s^−1^, 379- and 417-nm wavelengths had strong lethal effects, resulting in 85% and 66% total mortalities (84% and 66% larval mortalities), respectively (Fig. 2; Supplementary Fig. S1). However, at 10 × 10^18^ photons·m^−2^·s^−1^, these two wavelengths resulted in 100% larval mortalities (Supplementary Fig. S1). The 438-nm blue-light wavelength also had a strong lethal effect at 10 × 10^18^ photons·m^−2^·s^−1^, showing 95% larval and total mortalities, although it did not have a significant lethal effect at 5 × 10^18^ photons·m^−2^·s^−1^. Irradiation with 407-nm and 454-nm blue-light wavelengths at 10 × 10^18^ photons·m^−2^·s^−1^ had almost similar lethal effects resulting in 72% and 66% total mortalities (72% and 65% larval mortalities), respectively. The 467-nm blue light resulted in a significant lethal effect, with 42% total and 36% larval mortality, only at 10 × 10^18^ photons·m^−2^·s^−1^. The 497-nm blue light, however, produced no significant lethal effect even at 10 × 10^18^ photons·m^−2^·s^−1^. The mortality under LL was similar to that irradiated with 497-nm blue light at 10 × 10^18^ photons·m^−2^·s^−1^ (Fig. 2; Supplementary Fig. S1). Irradiation with blue light to larval feed did not influence the larval and pupal mortalities (Fig. S2).

### Lethal effect of blue-light irradiation on Ae. albopictus pupae

**Figure 3:**
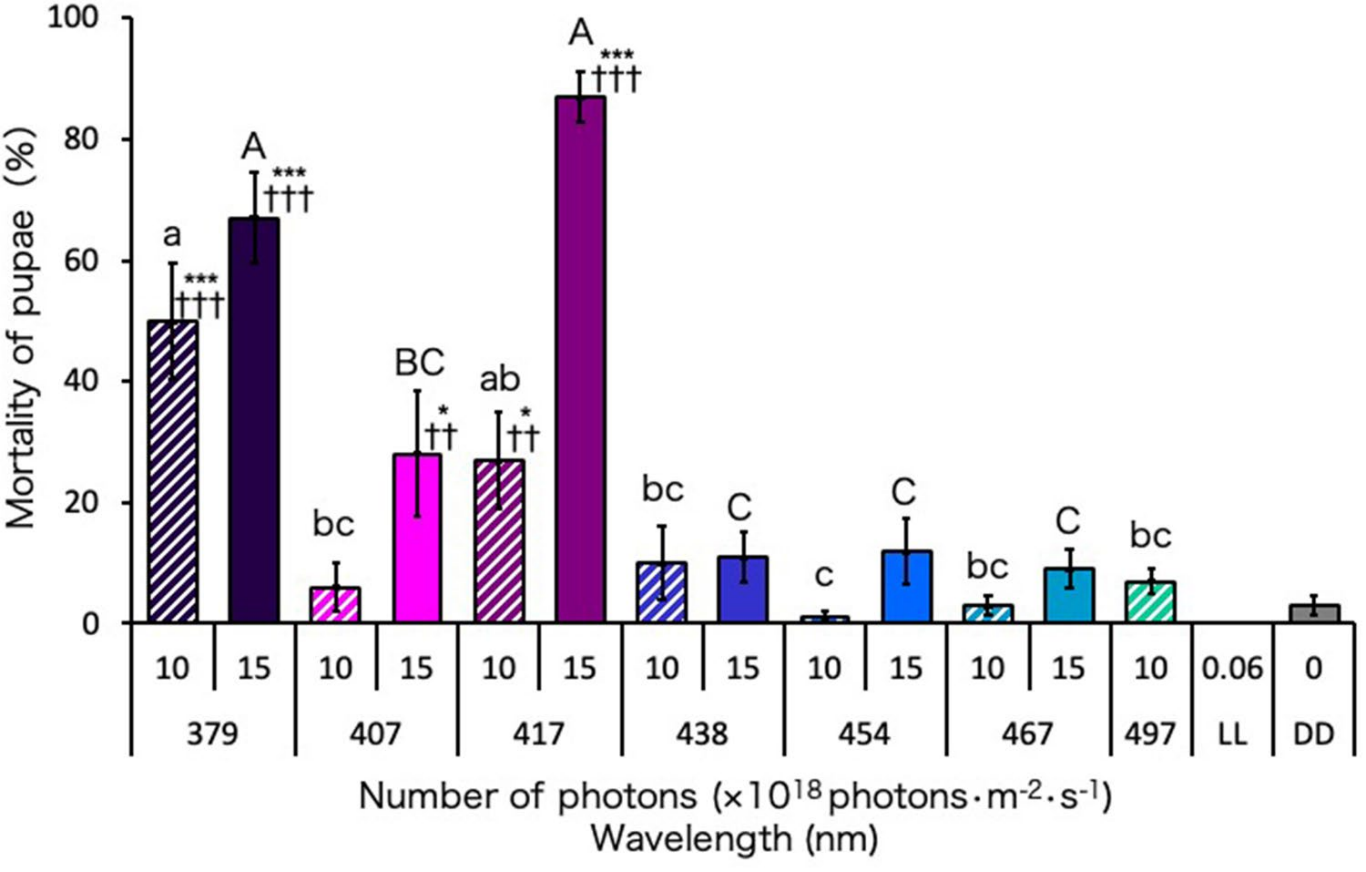
Mortality of *Aedes albopictus* irradiated with blue light during the pupal stage. Data represent the means ± standard errors. Asterisks above the bars indicate significant differences between the treatments (UVA and blue light irradiation) and control [dark condition (DD)] (Steel test: *p < 0.05, ***p < 0.001). Daggers above the bars indicate significant differences between the treatments (UVA and blue light irradiation) and the control [continuous white light condition (LL)] (Steel test: ^††^*p* < 0.01, ^†††^*p* < 0.001). Bars with the same letters are not significantly different (Steel–Dwass test, *p* > 0.05). Ten replications (10 pupae per replicate) were conducted.

Irradiation with the 417-nm blue light produced the strongest lethal effect on the pupae among the tested blue-light wavelengths (Fig. 3). At 15 × 10^18^ photons·m^−2^·s^−1^, the mortality of the pupae irradiated with the 417-nm blue light (87% mortality) was higher than that of the pupae irradiated with the 379-nm UVA light (67% mortality), whereas at 10 × 10^18^ photons·m^−2^·s^−1^, the mortality obtained with 379-nm wavelength (50% mortality) was higher than that obtained with 417-nm wavelength (27% mortality). Only the 417-nm blue light had a significant lethal effect at 10 × 10^18^ photons·m^−2^·s^−1^ among the tested blue-light wavelengths. The 407-nm blue light also had a significant lethal effect with only 28% mortality at 15 × 10^18^ photons·m^−2^·s^−1^. The other blue-light wavelengths had no significant lethal effect even at 15 × 10^18^ photons·m^−2^·s^−1^.

### Lethal effect of blue-light irradiation on Ae. albopictus adults

**Figure 4:**
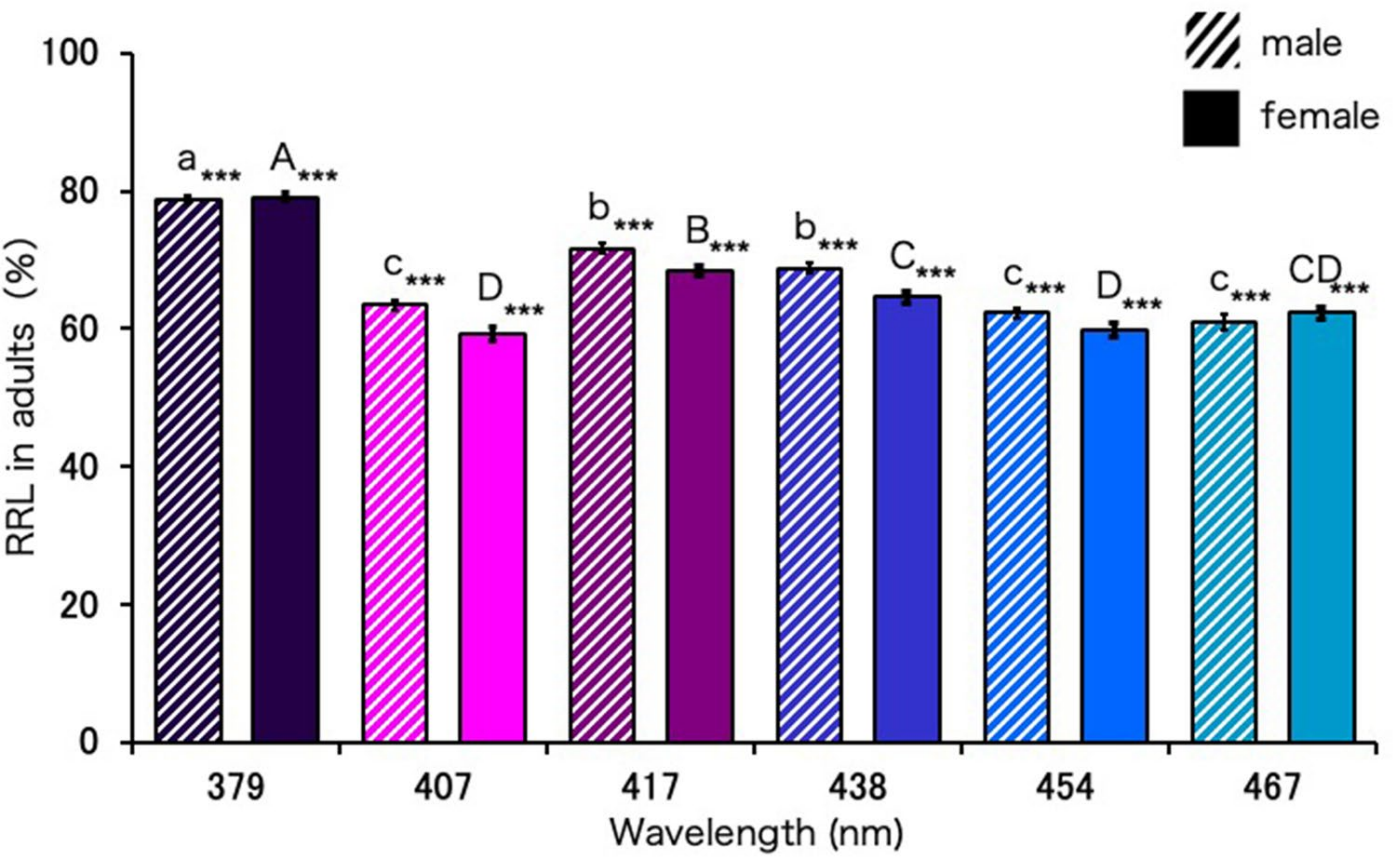
Reduction rate of longevity (RRL) of *Aedes albopictus* adults irradiated with blue light. Data represent the means ± standard errors. Asterisks above the bars indicate significant differences between the treatments (UVA and blue light irradiation) and control [dark condition (DD)] (Steel test, ***p < 0.001). Bars with the same letters are not significantly different (Steel–Dwass test, *p* > 0.05). Fifty replications (5 adults × 10 Petri dishes) were conducted.

The mean adult longevity values of males and females kept under DD were 36.02 and 47.28 days and those kept under LL were 25.40 days and 38.14 days, respectively (Supplementary Fig. S3). In contrast, the mean adult longevity values of males and females irradiated with UVA or blue light were 7.62–14.06 and 9.88–19.26 days, respectively, being significantly different from those under LL and DD. The reduction rate of longevity (RRL) values of the males and females irradiated with the 379-nm UVA light (78.85% and 79.10%, respectively) were the highest followed by the RRL values of those irradiated with the 417-nm (71.68% and 68.40%, respectively) and 438-nm blue-light wavelengths (68.74% and 64.59%, respectively) (Fig. 4). The RRL values of males and females irradiated with the 407-nm (63.46% and 59.26%, respectively), 454-nm (62.30% and 59.86%, respectively), and 467-nm wavelengths (60.97% and 62.35%, respectively) were almost similar.

## Discussion

Continuous white-light exposure (LL) showed no lethal effect on the larvae and pupae of *Ae. albopictus*. Therefore, the lethal effects of UVA and blue light on them presumably did not result from the disturbance of circadian rhythms. On the other hand, longevities of adults under LL were shorter than those under DD. The reduction in adult longevity under LL compared with that under DD may be attributed to the disturbance of circadian rhythms. However, the longevities of adults irradiated with UVA or blue light were shorter than those under LL. These results indicate that UVA and blue-light irradiation are lethal to the adults as well as the larvae and pupae.

In some insects, such as *Drosophila melanogaster* and *Galerucella grisescens*, the toxic blue-light wavelengths change with growth^2,8^. In contrast, the present study showed that, in *Ae. albopictus*, the most effective wavelength did not vary among developmental stages, in a manner similar to *C. pipiens* f. *molestus*^1,6^. Moreover, in our previous studies, we reported that effective blue-light wavelengths are species-specific in insects^1,2,6^. In *Ae. albopictus*, the most toxic blue-light wavelength for all stages, except for eggs, was the same as that for *C. pipiens* f. *molestus*, i.e. ~ 420 nm.

In our previous study, we indicated that reactive oxygen species (ROS) influence the lethal effect of blue light^8^. In mammals, the ROS generated by blue-light irradiation damage their retina cells^36,37^. Therefore, we speculated that the ROS produced by the absorption of specific blue-light wavelengths in insect tissues damages them and kills the insects^1^. It is likely that the absorption mechanism of the blue light having a lethal effect is common to *C. pipiens* f. *molestus* and *Ae. albopictus* through all developmental stages except for eggs. For example, mitochondria are known to specifically absorb ~ 420-nm light, and this absorption is attributed to the presence of porphyrins^38^. In addition, it has been reported that the absorption of wavelengths of ~ 420 nm by porphyrins interacts with triplet oxygen molecules to form radicals and ROS, leading to cell death^39–43^. The high lethal effect of ~ 420 nm on *Ae. albopictus* may be caused by the ROS produced by absorption of this wavelength of blue light by mitochondria.

Unlike the findings on *C. pipiens* f. *molestus* eggs, no blue-light wavelength exhibited a lethal effect on the eggs of *Ae. albopictus*. The difference in lethal effects on the eggs of both species may be related to the colours of their eggs. The eggshell colour of *C. pipiens* f. *molestus* is light brown, whereas that of *Ae. albopictus* is black. Therefore, the blue light is thought to easily reach the inner tissues of the eggs through the eggshell in *C. pipiens* f. *molestus* but not in *Ae. albopictus*. Furthermore, the mortalities of *Ae. albopictus* eggs irradiated with the blue-light wavelengths of 407–467 nm were significantly lower at the photon density of 10 × 10^18^ photons·m^−2^·s^−1^ than under DD. On the other hand, there was no significant difference in egg mortalities between LL and 407- to 467-nm blue-light irradiation. The cause of the improvement of the survival rate of eggs irradiated with blue light or LL remains unclear. The presence of adequate light conditions (photon density and wavelength) may have a positive effect on the growth of *Ae. albopictus* eggs.

Our findings indicate that immature populations of *Ae. albopictus* may be reduced by irradiation with ~ 420-nm blue light. Irradiation with 417-nm blue light at 10 × 10^18^ photons·m^−2^·s^−1^ resulted in 100% mortality in the larvae of *Ae. albopictus*. Therefore, *Ae. albopictus* adult populations can be reduced if the larvae are reliably exposed to ~ 420-nm blue light at photon densities higher than 10 × 10^18^ photons·m^−2^·s^−1^. *Aedes albopictus* can take advantage of any natural or artificial water reserve, and the eggs can withstand long-term drying in the diapause phase^44^. Water-holding containers, such as tyres, plastic buckets, plastic drums, and flowerpots, are their preferred field habitat^45,46^. We can prevent the immature stages of the mosquitoes from inhabiting these containers by removing water from them or by covering the containers with sheets. However, the mosquitoes also inhabit artificial waterbodies, such as ditches, reservoirs, and ponds in cemeteries, residential sites, and parks^45,47^, where it is difficult to drain water. The lethal effect of blue light may be useful for reducing mosquito populations in these sites. Irradiation of the water surface of artificial waterbodies using ~ 420-nm wavelength LED or laser diode (LD) devices (e.g. panel light type, tube light type, and floodlight type devices) could presumably be a practical application of the lethal effect of blue light on *Ae. albopictus*. Mosquito control using blue light is considered a highly safe and eco-friendly alternative to insecticides. The toxicity of blue light to biological organisms is much lower than that of UV, and blue-light irradiation generates no residuals. However, blue light irradiation induces injury in mammalian retina cells as mentioned above. Therefore, it is necessary that the installation is performed appropriately such that humans are prevented from directly looking at the light source. In addition, it is necessary to place the light source such that the negative effect on non-target organisms is prevented as well.

The distance between light source and mosquitoes in this study was ~ 200 mm. However, the distance under the field conditions is likely to be greater than that in the laboratory. The values of photon flux densities on irradiated surfaces are important for the lethal effect on insects. Therefore, it is necessary to design light sources (e.g. the characteristics of light distribution and LED output power) that can irradiate target insects at higher than 10 × 10^18^ photons·m^−2^·s^−1^ from the distance required for practical use. In addition, it is necessary to confirm the lethal effect of light source designs in a semi-field test.

*Aedes albopictus* is one of the most widespread Diptera species in the world^44^. They occur in urban areas as well as rural areas^47^ and transmit various arboviruses^23^. The worldwide distribution of arboviruses, such as dengue, chikungunya, and Zika viruses, is expanding because of globalised traffic (e.g. air passengers) and trade^48,49^. Therefore, in many regions, the outbreak risks of these diseases are expected to increase further in the future partly because of the impact of climate change. The lethal effect of blue light on mosquitoes, if put into practical use, will be able to contribute to the control of the outbreak of these diseases.

## Methods

### Insects

Fifty adult females of *Ae. albopictus* were collected from Nishitokyo (35.43° N, 139.32° E, Tokyo, Japan) to establish a colony that was maintained in our laboratory (Graduate School of Agricultural Science, Tohoku University, Sendai, Japan). Eggs, larvae, and pupae were reared in a plastic container (150 mm diameter × 91 mm height) containing 250 mL of tap water decalcified for 48 h, with a constant supply of fishery feed for trout juveniles (EC 1C, Feed One Co., Ltd., Yokohama, Japan). Adults were maintained in a mesh cage (300 mm × 300 mm × 300 mm) containing an Erlenmeyer flask (50 mL) and a plastic cup (30 mm diameter × 35 mm height). Cotton wool soaked with 10% honey solution (50 mL) was placed in the flask (food substitute), and a filter paper soaked with water was placed in the cup (oviposition substrate). Adult females were fed human blood by offering the author’s (Taniyama) arm every three days after five days of the emergence. The blood was given to mosquitoes until they left the arm. The blood of only Taniyama was given to the mosquitoes to avoid the influence of different blood on the survival of eggs and larvae. The oviposition substrate was placed in the cage five days after the first sucking of blood. Subsequently, the oviposition substrate with eggs deposited by females was taken out from the cage after five days and put into the water in the above-mentioned plastic container. In the cage, fresh oviposition substrate was placed. All stages were maintained in a constant-temperature room at 25 ± 1 °C and ~ 60% relative humidity under a 16 h:8 h (light:dark) photoregime. The offspring from more than six generations of the above colony were used for the present study.

### LED light irradiation

LED lighting units (IS-mini®, ISL-150 × 150 Series; CCS Inc., Kyoto, Japan; light emission surface: 150 mm × 150 mm; arrangement: 360 LEDs were equally arranged on a panel; LED type: φ 3 mm plastic mould) with power supply units (ISC-201–2; CCS Inc., Kyoto, Japan) were used for light irradiation. Insects were irradiated with LED light in a multi-room incubator (LH-30CCFL-8CT; Nippon Medical & Chemical Instruments Co., Ltd., Osaka, Japan). The emission spectra and the number of photons (photons·m^−2^·s^−1^) were measured using a high-resolution spectrometer (HSU-100S; Asahi Spectra Co., Ltd., Tokyo, Japan; numerical aperture of the fibre: 0.2) in a dark room. The number of photons was adjusted by current control using a power supply unit. During measurements, the distance between the LED lighting unit and the spectrometer sensor was set to be approximately equal to the same distance as between the insects and the LED lighting unit in the incubator. Because the mosquitoes were irradiated through a glass plate or glass lid, the same plate or lid was placed between the light source and sensor during measurement. The number of photons was measured five times before and after each experiment. The average values of the number of photons in each experiment are shown in Supplementary Tables S1–S4.

### Lethal effect of blue-light irradiation on Ae. albopictus eggs

Eggs were collected from the stock cultures within 1 h of deposition and were placed (n = 20) in water (12 mL) in a glass Petri dish (50 mm diameter × 15 mm height). The Petri dish was placed in a multi-room incubator (25 ± 1 °C) and irradiated with LED light at wavelengths of 379, 407, 417, 438, 454, 467, and 497 nm with set values of 10 × 10^18^ or 15 × 10^18^ photons·m^−2^·s^−1^ for 20 days. The distance between the light source and eggs (i.e. the water surface in the Petri dish) was 214 mm. The number of hatchlings (larvae) was counted every day to determine egg mortality. The hatchlings were removed from the Petri dish every day. In the control treatment, the eggs were maintained under LL {i.e. continuous white light condition with a cold cathode fluorescent lamp (CCFL, wavelength range: 250–1000 nm, photon flux density: 0.06 × 10^18^ photons·m^−2^·s^−1^, Nippon Medical & Chemical Instruments Co., Ltd., Osaka, Japan)} and DD (i.e. no irradiation) for 20 days, and egg mortality was determined as described above. In the LL control, the mosquitoes were irradiated at 0.06 × 10^18^ photons·m^−2^·s^−1^ because this is the maximum photon flux density produced by the CCFL. The photon flux density of the CCFL used for the control was much lower than those of the LEDs. However, white light contains UV and blue light because it comprises a wide range of wavelengths (250–1000 nm). If mosquitoes are irradiated with white light at a high photon density, they will be killed by the UV and blue light contained in white light. Therefore, the photon flux density used for LL conditions is adequate for investigating whether disruption of the circadian rhythm by continuous light conditions (not UV and blue light energy) affects the lifespan of an insect. The average number of photons and emission spectra of the CCFL are shown in Supplementary Tables S1–S4 and Supplementary Figure S4, respectively. Ten replications (dishes) were performed for each dose of each light wavelength.

### Lethal effect of blue-light irradiation on the larval to pupal stages of Ae. Albopictus

The method used to irradiate larval to pupal stages was the same as that in our previous study^6^. Hatchlings were collected from the stock cultures within 12 h of hatching and were placed (n = 10) in water (50 mL) in polyethylene terephthalate (PET) ice-cream cups (60 mm diameter × 38 mm height, Risupack, Co., Ltd., Aichi, Japan). Fishery feed for trout juveniles (EC 1C, Feed One Co., Ltd., Yokohama, Japan) (0.01 g) was supplied in the cups on the first day of irradiation, and subsequently, 0.03 g was added five days after the start of the irradiation. Rearing protocols for *Aedes* mosquitoes recommend feeding small quantities of food every 24–48 h to avoid mortality due to either biofilm development or starvation. However, feeding interrupts continuous irradiation. Therefore, the above-mentioned feeding methods were adopted in this study after an investigation of the appropriate feeding intervals and the amount of food required to avoid biofilm development or starvation. The cups were each covered with a glass plate, placed in the multi-room incubator (25 ± 1 °C), and irradiated with LED light at the same wavelengths as mentioned above, with set values of 5 × 10^18^ or 10 × 10^18^ photons·m^−2^·s^−1^. The distance between the light source and the larvae or pupae (i.e. the water surface in the cup) was 203 mm. The larvae in the cups were irradiated until they had all died or developed to the adult stage. The number of pupated larvae and emerging adults were counted to determine the larval and pupal mortalities, respectively. In the control treatment, mosquitoes were maintained under LL and DD until they had all died or developed to the adult stage, after which the larval and pupal mortalities were determined using the same method as described above. Ten replications (cups) were performed for each dose of each light wavelength.

The contribution of blue-light exposed feed to larval mortality was also investigated. The fishery feed for trout juveniles (0.01 g) in a cup with 50 ml of water was irradiated with 417-nm light at set values of 10 × 10^18^ photons·m^−2^·s^−1^ for 3 days. After the irradiation, ten hatchlings within 12 h after hatching were placed in the cup, and were maintained under DD. After three days, the larvae were moved to a new cup with 0.03 g feed irradiated with 417-nm light at the same photon flux density for five days. Each of the cups were covered with a glass plate and placed in a multi-room incubator (25 ± 1 °C). All larvae in the cups were maintained under DD until all of them either died or developed to the adult stage. The number of pupated larvae and emerged adults were counted to determine the larval and pupal mortalities, respectively. Five replications (cups) were performed.

### Lethal effect of blue-light irradiation on Ae. albopictus pupae

Pupae were collected from the stock cultures within 4 h of pupation and were placed (n = 10) in water (50 mL) in PET ice-cream cups (60 mm diameter × 38 mm height). The cups were each covered with a glass plate, were placed in the multi-room incubator (25 ± 1 °C), and irradiated with LED light at the same wavelengths as mentioned above with the set values of 10 × 10^18^ or 15 × 10^18^ photons·m^−2^·s^−1^. The distance between the light source and the pupae (i.e. the water surface in the cup) was 203 mm. The pupae in the cups were irradiated until they had all died or developed to the adult stage. The number of emerging adults were counted to calculate the pupal mortalities. In the control treatment, the pupae were maintained under LL and DD until they had all died or developed to the adult stage, after which the pupal mortalities were determined using the above-described method. Ten replications (cups) were performed for each dose of each light wavelength.

### Lethal effect of blue-light irradiation on Ae. albopictus adults

The method used to irradiate adults was the same as that in our previous study^6^. Adults were collected from the plastic rearing containers within 12 h of emergence, and their pairs (n = 5 pairs) were released onto circular cotton pads (10 g, diameter 90 mm) soaked with 5% honey solution (10 mL) in a glass Petri dish (60 mm diameter × 90 mm height). The Petri dish was placed in a multi-room incubator equipped with an LED lighting unit. The distance between the light source and the adults (i.e. the bottom of the Petri dish) was 225 mm. The adults were irradiated with different wavelengths of light at 25 ± 1 °C until all of them died. We counted the number of dead adults every day and replaced the Petri dish containing the honey water every 2 days. Lethal effects of irradiation at the set values of 15 × 10^18^ photons·m^−2^·s^−1^ were compared among the six wavelengths [379, 407, 417, 438, 454, and 467 nm]. In the test for adults, a high lethal effect was not obtained even at 15 × 10^18^ photons·m^−2^·s^−1^; therefore, the effect at 10 × 10^18^ photons·m^−2^·s^−1^ was not investigated. In the control treatment, the longevities of the mosquitoes maintained under LL and DD (no irradiation) were determined. The lethal effect was determined using the RRL values, which were calculated as follows:

$${\text{RRL }} = { 1}00 \, \left( {{\text{LD }}{-}{\text{ LI}}} \right)/{\text{LD}}$$

where LD and LI represent the average longevities of nonirradiated (DD) and irradiated adults, respectively. Fifty replications (five pairs adults × 10 Petri dishes) were performed for each dose of each light wavelength.

### Statistical analysis

Comparisons of mortalities and RRLs between irradiation and control treatments and among the studied wavelengths were analysed using the Steel and Steel–Dwass tests, respectively. The investigation of the contribution of blue-light exposed feed to larval mortality was performed using the Mann–Whitney *U* test. The calculations were performed using R version 4.0.2^50^.

### Ethical approval

This study is exempt from the need for ethical approval under local law as per advice received from the Ethical Review Committee, Graduate School of Agricultural Science, Tohoku University.

## Supplementary Information

Supplementary Information.

## Data Availability

The datasets are available from the corresponding author on a reasonable request.
