# Supplementary material for: Lethal effect of blue light on Asian tiger mosquito, Aedes albopictus (Diptera: Culicidae)
Source: Sci Rep. 2022 Jun 16;12:10100. doi: 10.1038/s41598-022-14096-y (PMC9203503; doi:10.1038/s41598-022-14096-y)
Supplement: Supplementary file 1 — Supplementary Information. [file 41598_2022_14096_MOESM1_ESM.docx]

Lethal effect of blue light on Asian tiger mosquito, *Aedes albopictus* (Diptera: Culicidae)

Katsuya Taniyama^1^, Masatoshi Hori^1*^

^1^Graduate School of Agricultural Science, Tohoku University, Sendai, Miyagi 980-8572, Japan

^*^Corresponding author: masatoshi.hori.a3@tohoku.ac.jp


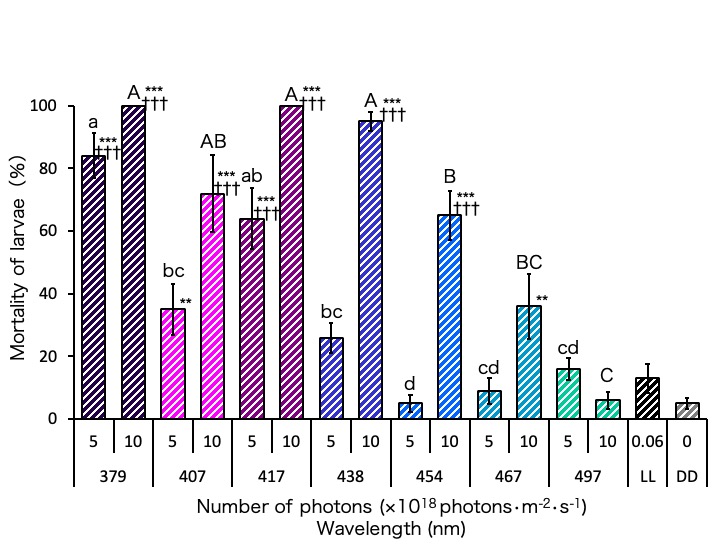


Supplementary Figure S1. Larval mortality of *Aedes albopictus* irradiated with blue light during the larval stage. Data represent the means ± standard errors. Asterisks above the bars indicate significant differences between the treatments (UVA and blue light irradiation) and control [dark condition (DD)] (Steel test: ^**^ *p* < 0.01, ^***^ *p* < 0.001). Daggers above the bars indicate significant differences between the treatments (UVA and blue light irradiation) and the control [continuous white light condition (LL)] (Steel test: ^†^*p* < 0.05). Bars with the same letters are not significantly different (Steel–Dwass test, *p* > 0.05). Ten replications (10 larvae per replicate) were conducted.


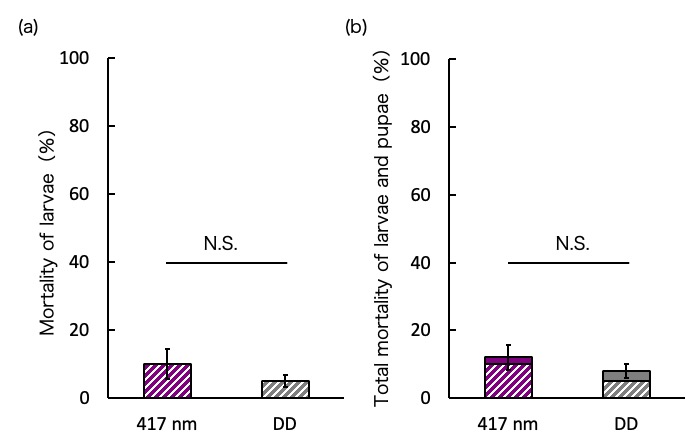


Supplementary Figure S2. Mortality of *Aedes albopictus* fed with fishery feed and irradiated with 417-nm blue light. Data represent the means ± standard errors. N.S. above the bars indicate no significant difference between 417-nm irradiation and the dark condition (DD) (Mann–Whitney *U* test, *p* > 0.05). Five replications (10 larvae per replicate) were conducted. (a) Mortality in the larval stage. (b) Total mortality in the larval and pupal stages.


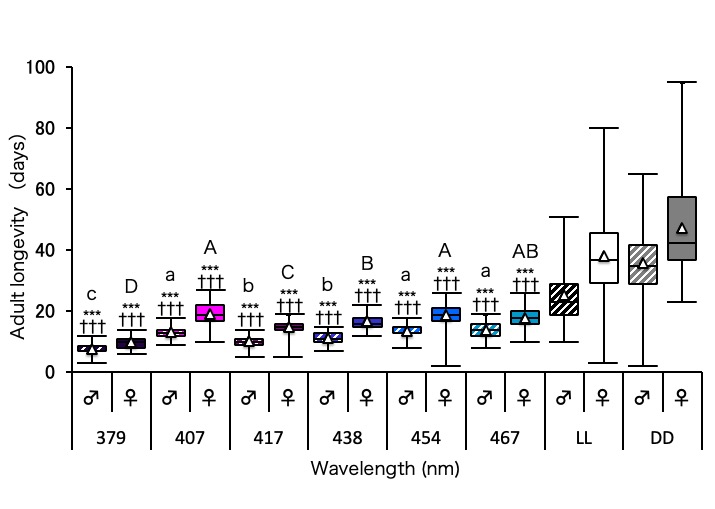


Supplementary Figure S3. Adult longevity of *Aedes albopictus* irradiated with blue light during the adult stage. Longevity of *Ae. albopictus* males (diagonal striped boxes) and females (solid boxes). The top and bottom bars indicate the maximum and minimum longevities observed in this experiment. The top and bottom of each box indicate the 75th and 25th percentiles, respectively. The internal line and open triangle are the median and mean values, respectively. In all wavelengths, significant differences were obtained between the treatments (UVA and blue light irradiation) and control [dark condition (DD)] (Steel test: ^***^ *p* < 0.001). Daggers above the bars indicate significant differences between the treatments (UVA and blue light irradiation) and the control [continuous white light condition (LL)] (Steel test: ^†††^*p* < 0.001). Box plots with the same letters are not significantly different (Steel–Dwass test, *p* > 0.05). Fifty replications (5 adults × 10 Petri dishes) were conducted for each dose of each light wavelength for each sex.


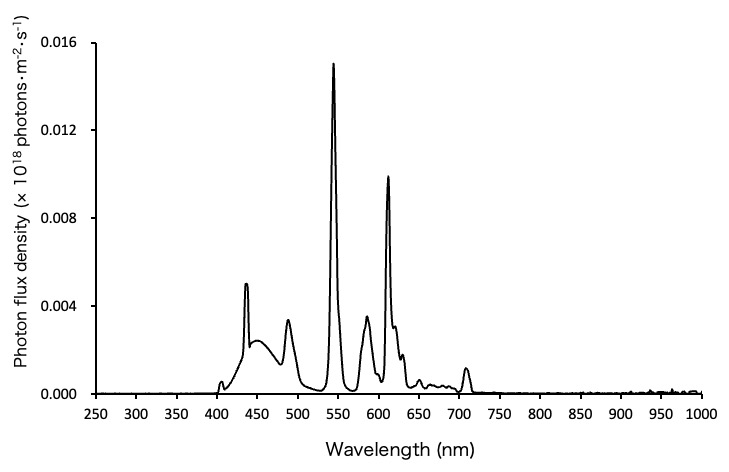


Supplementary Figure S4. Emission spectra of the cold cathode fluorescent lamp (CCFL) used in the LL controls.

Supplementary Table S1. Actual measurement values of the photon flux density in the experiment conducted to measure the lethal effect of blue-light irradiation during the egg stage of *Aedes albopictus*

| Wavelength (nm) | Mean actual measured values ± standard error (× 10^18^ photons･m^-2^･s^-1^)^*^ | |
| --- | --- | --- |
|  | Set values (× 10^18^ photons･m^-2^･s^-1^) | |
|  | 10 | 15 |
| 379 | 9.77 ± 0.06 | 14.42 ± 0.15 |
| 407 | 9.93 ± 0.02 | 14.97 ± 0.01 |
| 417 | 9.92 ± 0.01 | 14.87 ± 0.03 |
| 438 | 9.92 ± 0.01 | 15.15 ± 0.03 |
| 454 | 9.96 ± 0.01 | 15.09 ± 0.06 |
| 467 | 9.94 ± 0.01 | 15.17 ± 0.06 |
| 497 | 9.55 ± 0.16 |  |
| LL (CCFL) | 0.06 ± 0.00 | |

^*^Mean value of the measurements conducted five times before and after each irradiation test

Supplementary Table S2. Actual measurement values of the photon flux density in the experiment conducted to measure the lethal effect of blue-light irradiation during the larval to pupal stages of *Aedes albopictus*

| Wavelength (nm) | Mean actual measured values ± standard error (× 10^18^ photons･m^-2^･s^-1^)^*^ | |
| --- | --- | --- |
|  | Set values (× 10^18^ photons･m^-2^･s^-1^) | |
|  | 5 | 10 |
| 379 | 5.01 ± 0.01 | 9.58 ± 0.11 |
| 407 | 5.03 ± 0.01 | 10.12 ± 0.05 |
| 417 | 5.13 ± 0.03 | 10.29 ± 0.12 |
| 438 | 4.83 ± 0.06 | 9.97 ± 0.02 |
| 454 | 4.90 ± 0.04 | 9.96 ± 0.02 |
| 467 | 5.01 ± 0.00 | 9.99 ± 0.02 |
| 497 | 5.04 ± 0.01 | 9.54 ± 0.12 |
| LL (CCFL) | 0.06 ± 0.00 | |

^*^Mean value of the measurements conducted five times before and after each irradiation test

Supplementary Table S3. Actual measurement values of the photon flux density in the experiment conducted to measure the lethal effect of blue-light irradiation during the pupal stage of *Aedes albopictus*

| Wavelength (nm) | Mean actual measured values ± standard error (× 10^18^ photons･m^-2^･s^-1^)^*^ | |
| --- | --- | --- |
|  | Set values (× 10^18^ photons･m^-2^･s^-1^) | |
|  | 10 | 15 |
| 379 | 9.92 ± 0.02 | 14.76 ± 0.05 |
| 407 | 10.08 ± 0.02 | 14.90 ± 0.03 |
| 417 | 10.02 ± 0.02 | 14.72 ± 0.05 |
| 438 | 10.00 ± 0.01 | 14.91 ± 0.02 |
| 454 | 10.05 ± 0.03 | 15.03 ± 0.01 |
| 467 | 10.13 ± 0.02 | 14.92 ± 0.01 |
| 497 | 10.02 ± 0.01 |  |
| LL (CCFL) | 0.06 ± 0.00 | |

^*^Mean value of the measurements conducted five times before and after each irradiation test

Supplementary Table S4. Actual measurement values of the photon flux density in the experiment conducted to measure the lethal effect of blue-light irradiation during the adult stage of *Aedes albopictus*

| Wavelength (nm) | Mean actual measured values ± standard error (× 10^18^ photons･m^-2^･s^-1^)^*^ |
| --- | --- |
|  | Set values (× 10^18^ photons･m^-2^･s^-1^) |
|  | 15 |
| 379 | 14.38 ± 0.11  14.92 ± 0.03  14.95 ± 0.02  14.97 ± 0.04  14.83 ± 0.03  15.02 ± 0.03 |
| 407 |  |
| 417 |  |
| 438 |  |
| 454 |  |
| 467 |  |
| LL (CCFL) | 0.06 ± 0.00 |

^*^Mean value of the measurements conducted five times before and after each irradiation test
